# Supplementary material for: Specialized post-arterial capillaries facilitate adult bone remodelling
Source: Nat Cell Biol. 2024 Nov 11;26(12):2020–34. doi: 10.1038/s41556-024-01545-1 (PMC11628402; doi:10.1038/s41556-024-01545-1)
Supplement: Supplementary file 1 — Supplementary Fig. 1. Analysis of published bone EC scRNA-seq datasets a–d, t-SNE (a–c) or UMAP (d) visualization of selected marker genes in different datasets, using colour-coding based on population labels from the original studies. Cdh5 marks all bone ECs and cells expressing type rECs markers Cav1, Dach1 and C1qtnf9 are indicated by red arrowheads. [file 41556_2024_1545_MOESM1_ESM.pdf]

nature cell biology

Supplementary information https://doi.org/10.1038/s41556-024-01545-1

Specialized post-arterial capillaries facilitate adult bone remodelling

In the format provided by the authors and unedited

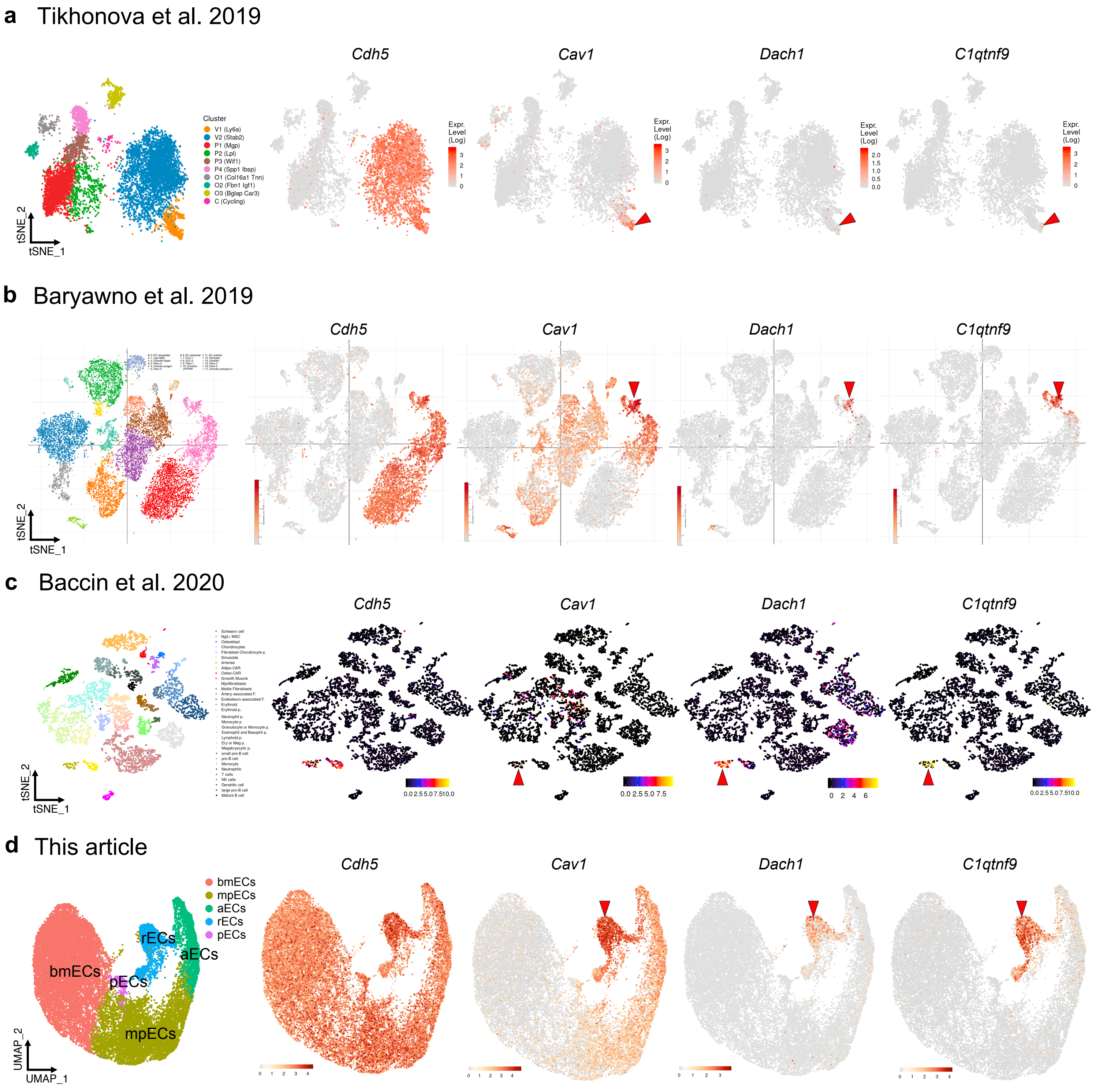

Supplementary Figure 1. Analysis of published bone EC scRNA-seq datasets a-d, tSNE (a-c) or UMAP (d) visualization of selected marker genes in different datasets, using color-coding based on population labels from the original studies. Cdh5 marks all bone ECs and cells expressing type rECs markers Cav1, Dach1, and C1qtnf9 are indicated by red arrowheads.
