# Supplementary material for: Specialized post-arterial capillaries facilitate adult bone remodelling
Source: Nat Cell Biol. 2024 Nov 11;26(12):2020–34. doi: 10.1038/s41556-024-01545-1 (PMC11628402; doi:10.1038/s41556-024-01545-1)
Supplement: Supplementary file 2 — Reporting Summary [file 41556_2024_1545_MOESM2_ESM.pdf]

nature portfolio

Corresponding author(s): Ralf H. Adams

Last updated by author(s): Sep 5, 2024

Reporting Summary

Nature Portfolio wishes to improve the reproducibility of the work that we publish. This form provides structure for consistency and transparency in reporting. For further information on Nature Portfolio policies, see our Editorial Policies and the Editorial Policy Checklist.

Statistics

For all statistical analyses, confirm that the following items are present in the figure legend, table legend, main text, or Methods section.

n/a	Confirmed	
☐	☒	The exact sample size (*n*) for each experimental group/condition, given as a discrete number and unit of measurement
☐	☒	A statement on whether measurements were taken from distinct samples or whether the same sample was measured repeatedly
☐	☒	The statistical test(s) used AND whether they are one- or two-sided<br>*Only common tests should be described solely by name; describe more complex techniques in the Methods section.*
☐	☒	A description of all covariates tested
☐	☒	A description of any assumptions or corrections, such as tests of normality and adjustment for multiple comparisons
☐	☒	A full description of the statistical parameters including central tendency (e.g. means) or other basic estimates (e.g. regression coefficient) AND variation (e.g. standard deviation) or associated estimates of uncertainty (e.g. confidence intervals)
☐	☒	For null hypothesis testing, the test statistic (e.g. *F*, *t*, *r*) with confidence intervals, effect sizes, degrees of freedom and *P* value noted<br>*Give P values as exact values whenever suitable.*
☒	☐	For Bayesian analysis, information on the choice of priors and Markov chain Monte Carlo settings
☒	☐	For hierarchical and complex designs, identification of the appropriate level for tests and full reporting of outcomes
☒	☐	Estimates of effect sizes (e.g. Cohen's *d*, Pearson's *r*), indicating how they were calculated

Our web collection on statistics for biologists contains articles on many of the points above.

Software and code

Policy information about availability of computer code

Data collection	RNA sequencing by NestSeq500, Confocal imaging were collected from Zeiss LSM780, Yokogawa Confocal microscope and LSM980; Cell sorting and analyzed by FACS Aria II cell sorter with FACSDiva (8.0.2) software (BD Bioscience)
Data analysis	No custom software or algorithm was used in this study. All software used in this study for data analysis are either commercially available or open source.<br>Data analysis and statistical tests were done using GraphPad Prism (v8 and v10).<br>Confocal images were analyzed with Volocity 6.3 (Quorum Techonology) or ImageJ2/Fiji (1.53u)<br>FACS data was processed with FlowJo (10.8.1.)<br>GraphPad Prism (v8 and v10) was used for statistical analyses. Figures were generated with Adobe illustrator 2020, 2021, 2024.<br><br>Single cell RNA sequencing data analysis<br>FASTQ-format sequencing raw data was initially processed using UMI-tools (version 1.1.2). The data was then aligned to the mouse reference genome (mm10) using STAR (version 2.7.10a), and quantified with Subread featureCounts (version 2.0.3) to generate an expression matrix. For bone stromal cells, the FASTQ-format sequencing raw data was processed with BD Rhapsody WTA Analysis pipeline (version 1.0) on SevenBridges Genomics online platform (SevenBridges) and expression matrix was used for further data analysis. Data normalization, dimensionality reduction and visualization were performed using Seurat package (version 4.3.0), unless otherwise specified. Plots plotted using Seurat and (https://github.com/SGDDNB/ShinyCell) ShinyCell.

For manuscripts utilizing custom algorithms or software that are central to the research but not yet described in published literature, software must be made available to editors and reviewers. We strongly encourage code deposition in a community repository (e.g. GitHub). See the Nature Portfolio guidelines for submitting code & software for further information.

Data

Policy information about availability of data

All manuscripts must include a data availability statement. This statement should provide the following information, where applicable:

- Accession codes, unique identifiers, or web links for publicly available datasets
- A description of any restrictions on data availability
- For clinical datasets or third party data, please ensure that the statement adheres to our policy

Sequencing data that supports the findings of this study have been deposited at Gene Expression Omnibus (GEO, https://www.ncbi.nlm.nih.gov/geo/) under the accession code GSE239627 (https://www.ncbi.nlm.nih.gov/geo/query/acc.cgi?acc=GSE239627). This is a SuperSeries record providing access to all related data, namely bone EC characterization and Dach1OE non-hematopoietic cells. The mouse reference genome (mm10; https://www.ncbi.nlm.nih.gov/datasets/genome/GCF_000001635.20/) was used for mapping reads in this study.
All other information supporting the findings of this study are available within this article or can be obtained from the corresponding author upon request.

Human research participants

Policy information about studies involving human research participants and Sex and Gender in Research.

Reporting on sex and gender	Sex and gender data was not disaggregated due to the small number of samples used in this study.
Population characteristics	NA
Recruitment	NA
Ethics oversight	Collection of human material was conducted with informed consent by patients/parents and with approval from the Swedish Ethical Research Authority and the National Board of Health and Welfare (ethical permissions numbers 2014/276-31/2 and 97-214).

Note that full information on the approval of the study protocol must also be provided in the manuscript.

Field-specific reporting

Please select the one below that is the best fit for your research. If you are not sure, read the appropriate sections before making your selection.

☒ Life sciences ☐ Behavioural & social sciences ☐ Ecological, evolutionary & environmental sciences

For a reference copy of the document with all sections, see nature.com/documents/nr-reporting-summary-flat.pdf

Life sciences study design

All studies must disclose on these points even when the disclosure is negative.

Sample size	No specific statistical methods were used to predetermine sample size. Sample size were chosen based on previous experience. (ref. Kusumbe et al. Nature 2014; Ramasamy et al., Nature 2014; Sivaraj et al., Elife 2020; Sivaraj et al., Cell Report 2021).
Data exclusions	To ensure data quality, cells were filtered based on the criteria: having a number of genes per cell (nFeature_RNA) between 500 and 6000, and a percentage of mitochondrial genes (percent.mito) less than 25. Additionally, genes were filtered to include only those present in a minimum of 3 cells. After filtering, the matrices were normalized using the NormalizeData function with the LogNormalize method and a scale factor of 10,000. Variable genes were identified using the FindVariableFeatures function, selecting the top 2000 genes with the variance stabilizing transformation (VST) method, while also excluding genes related to the cell cycle (GO:0007049).
Replication	All experiments were repeated at least three times with an exception for scRNAseq experiments. All other experiments performed independently to ensure reproducibility. All the attempts of replication experiments were successful.<br>scRNA-seq experiments were performed once with pooled animals for each age and treatment group. Integration of these samples led to robust and reliable results across conditions.
Randomization	No formal method of randomization was used. All experiments involving wildtype mice were performed using C57Bl6 strain female mice. For mutant studies we used both male and female of same age group, and phenotype were always compared between same age and sex of animals. scRNAseq of 3-week-old bone endothelial cells and Dach1OE scRNAseq utilized both male and female mice.
Blinding	Blinding was not be feasible due to logisitic requirements as well as cost and personnel constraints.

Reporting for specific materials, systems and methods

We require information from authors about some types of materials, experimental systems and methods used in many studies. Here, indicate whether each material, system or method listed is relevant to your study. If you are not sure if a list item applies to your research, read the appropriate section before selecting a response.

Materials & experimental systems

n/a	Involved in the study
☐	☒ Antibodies
☐	☒ Eukaryotic cell lines
☒	☐ Palaeontology and archaeology
☐	☒ Animals and other organisms
☒	☐ Clinical data
☒	☐ Dual use research of concern

Methods

n/a	Involved in the study
☒	☐ ChIP-seq
☐	☒ Flow cytometry
☒	☐ MRI-based neuroimaging

Antibodies

Antibodies used

All antibody details (clone and manufacturer) are included in methods and also listed below:
Antibody Manufacturer Catalog number Concentration
Primary antibodies used: rat monoclonal anti-Endomucin (V.7C7) (Santa Cruz, sc-65495, 1:100),
goat polyclonal anti-VEGFR3 (R&D Systems, AF743, 1:50),
rabbit polyclonal anti-Cav1 (Cell Signaling, 3238, 1:50),
chicken polyclonal anti-GFP (Abcam, ab13970, 1:200),
rabbit polyclonal anti-Osterix (Abcam, ab22552, 1:300),
rabbit monoclonal anti-Runx2 EPR14334 (Abcam, ab192256, 1:200),
rabbit monoclonal anti-Ki67 (Cell Signaling, 12202, 1:200),
rabbit monoclonal anti-vATPase (Abcam, ab200839, 1:200),
rabbit polyclonal anti-Collagen IV (AbD Serotec, 2150-1470, 1:100),
rabbit polyclonal anti-Collagen IIIA1 (Abcam, ab7778, 1:100),
goat polyclonal anti-Hif1a (R&D Systems, AF1935, 1:100),
rabbit polyclonal anti-Hif1a (Santa Cruz, sc-10790, 1:100),
rabbit monoclonal anti-GPI (Cell Signaling, 94068, 1:200),
rabbit monoclonal anti-CD73 (Cell Signaling, 13160, 1:200),
rabbit monoclonal anti-Hmox1 (Cell Signaling, 86806, 1:200),
rabbit polyclonal anti-Dach1 (Proteintech, 10914-1-AP, 1:200),
rabbit polyclonal anti-NG2 (Millipore, AB5320, 1:200),
goat polyclonal anti-Cxadr (R&D Systems, AF2654, 1:50),
rabbit monoclonal anti-Sox11 (Abcam, ab134107, 1:800),
rat monoclonal anti-Madcam1 (Abcam, ab80680, 1:100),
rabbit polyclonal anti-Fabp4 (Abcam, ab13979, 1:100),
mouse monoclonal anti-Mct4 (Santa Cruz, AF647, 1:100),
rabbit monoclonal anti-Glut1 (Cell Signaling, 12939, 1:100),
goat polyclonal anti-Dll4 (R&D Systems, AF1389, 1:50),
chicken polyclonal anti-Mct1 (Millipore, AB1286-I, 1:100),
rabbit polyclonal anti-Dach1 (Proteintech, 10914-1-AP, 1:500),
goat polyclonal anti-Cdh5 (R&D Systems, AF938, 1:100),
rat anti-CD31 (BD Pharmingen, 553370; 1:100),
goat anti-VEGFR2 (R&D Systems, AF644; 1:100),
Mouse anti-VEGFR3 (R&D Systems, MAB3491, 1:100),
Goat anti-VEGFR2 (R&D Systems, AF357; 1:100),
Anti-rat Alexa Fluor 488 (ThermoFisher Scientific, A21208, 1:100-1:300),
anti-rabbit Alexa Fluor 488 (ThermoFisher Scientific, A21206, 1:100-1:300),
anti-chicken Alexa Fluor 488 (Jackson Laboratories, 703-545-155, 1:100-1:300),
anti-goat Alexa Fluor 546 (ThermoFisher Scientific, A11056, 1:100-1:300),
anti-rabbit Alexa Fluor 546 (ThermoFisher Scientific, A10040, 1:100-1:300),
anti-rat Alexa Fluor 594 (ThermoFisher Scientific, A21209, 1:100-1:300),
anti-rabbit Alexa Fluor 594 (ThermoFisher Scientific, A21207, 1:100-1:300),
anti-goat Alexa Fluor 594 (ThermoFisher Scientific, A11058, 1:100-1:300),
anti-goat Alexa Fluor 647 (ThermoFisher Scientific, A21447, 1:100-1:300),
anti-rabbit Alexa Fluor 647 (ThermoFisher Scientific, A31573, 1:100-1:300),
anti-rat Alexa Fluor 647 (Jackson Laboratories, 712-605-153, 1:100-1:300),
anti-mouse Alexa Fluor 488 (Thermo Fisher Scientific, A21202, 1:500),
Cy™3 AffiniPure™ Goat Anti-Mouse IgG (Jackson ImmunoResearch, 115-165-003; 1:400),
anti-goat Alexa-647 (ThermoFisher Scientific, A32849; 1:400),
anti-rabbit Alexa Fluor 546 (Thermo Fisher Scientific, A10040, 1:500) for HUVECS
DAPI

Validation

All antibodies used in the study have been commercially available and previously used by our group (ref. kusumbe et al. Nature 2014;

Validation	Ramasamy et al., Nature 2014; Sivaraj et al., Elife 2020; Sivaraj et al., Cell Report 2021). The complete information and all validation information for each Ab as well as previous publications that have used each Ab can be found on the manufacturer's website.

Eukaryotic cell lines

Policy information about cell lines and Sex and Gender in Research

Cell line source(s)	primary Human Umbilical Vein Endothelial Cells (HUVECs) : Provitro<br>primary Human Mesenchymal Stem Cells (HMSCs) (Lonza, PT-2501)
Authentication	Authentication was performed for each cell lot by vendors and cell identity was confirmed by antibody staining of cultured cells.
Mycoplasma contamination	Mycoplasma contamination test results were negative.
Commonly misidentified lines (See ICLAC register)	There is no commonly misidentified lines.

Animals and other research organisms

Policy information about studies involving animals; ARRIVE guidelines recommended for reporting animal research, and Sex and Gender in Research

Laboratory animals	All animals used in this study are Mus musculus species, C57/BL6 background strain independent of genotype.<br><br>Flt4-CreERT2 lineage tracing experiments were performed at Uppsala University and approved by the Uppsala Laboratory Animal Ethical Committee. The remaining animals were housed in an animal facility at the Max Planck Institute for Molecular Biomedicine in specific pathogen-free conditions in individually ventilated cages (IVC) with a consistent light/dark cycle, free access to food and water, and controlled temperature and humidity. Mice were routinely genotyped by PCR using allele-specific primers.<br><br>Transgenic mice were generated from our laboratory, Cdh5(PAC)-CreERT2, Bmx-CreERT2 & Cdh5-mTomato-nGFP; Sp7-mcherry mice from Jackson Laboratory; Efnb2-H2BGFP mice from Philippe Soriano Laboratory; Aplnr-CreERT2, Rosa26-Dach1OE, Dach1 flox/flox mice from the Red-Horse Laboratory; Flt4-CreERT2 mice from Sagrario Ortega Laboratory; Dll4 flox/flox mice from Freddy Radtke Laboratory; Rosa26-mTG mice from Jackson Laboratory. 3-week old, 6-week old, 12-week old and 75-week-old mice were used for most of the experiments. Sex of the mice is indicated wherever necessary.<br><br>For mutant experiments, mice were bred to Aplnr-CreERT2 or Cdh5(PAC)CreERT2 to generate inducible mouse model. Cre negative were used as litter mate control. Most of the experiments were performed at P30 or 12 weeks.<br><br>All animal experiments were conducted following the 3Rs (replacement, reduction, and refinement) and in accordance with institutional guidelines and laws, following protocols (84-02.04.2016.A160, 81-02.04.2018.A171, 81-02.04.2020.A212, 81-02.04.2020.A416 and 81-02.04.2022.A198) approved by the Landesamt für Natur, Umwelt, and Verbraucherschutz of North Rhine-Westphalia, Germany.
Wild animals	No wild animals were used in the study.
Reporting on sex	Female mice were used for most of the experiments. Male and female mice were used in 3-week-old bone endothelial scRNAseq and Dach1OE scRNAseq at Postnatal day 30.
Field-collected samples	No field collected samples were used.
Ethics oversight	All the animal experiments described in this study were conducted following the 3Rs (replacement, reduction, and refinement). Mice were housed in specific pathogen-free conditions in individually ventilated cages (IVC) with a consistent light/dark cycle, food, water, and controlled temperature. Experiments were performed at the MPI for Molecular Biomedicine in accordance with institutional guidelines and laws, following protocols (84-02.04.2016.A160, 81-02.04.2018.A171, 81-02.04.2020.A212, 81-02.04.2020.A416 and 81-02.04.2022.A198) approved by the Landesamt für Natur, Umwelt, and Verbraucherschutz (LANUV) of North Rhine-Westphalia, Germany. Flt4-CreERT2 lineage tracing experiments were performed at Uppsala University and approved by the Uppsala Laboratory Animal Ethical Committee.

Note that full information on the approval of the study protocol must also be provided in the manuscript.

Flow Cytometry

Plots

Confirm that:

☒ The axis labels state the marker and fluorochrome used (e.g. CD4-FITC).

☒ The axis scales are clearly visible. Include numbers along axes only for bottom left plot of group (a 'group' is an analysis of identical markers).

☒ All plots are contour plots with outliers or pseudocolor plots.

☒ A numerical value for number of cells or percentage (with statistics) is provided.

Methodology

Sample preparation	All sample preparation methods are written in the method section and available here.<br><br>To enrich for bone endothelial cells, single-cell suspensions were prepared from Cdh5-mT/nG reporter femurs and tibias. First, the surrounding muscle tissue was carefully removed and the epiphysis was detached from both the femur and tibia. Next, the cleaned samples were then collected in a solution of collagenase types I and IV (2 mg/ml). The bones were cut into small pieces and crushed using mortar and pestle. After 20 min of digestion at 37 °C, the crushed samples were transferred to 70µm strainers in 50ml tubes to obtain a single–cell suspension.<br><br>The cell suspension was resuspended in blocking solution (1% BSA and 1mM EDTA in PBS without Ca2+/Mg2+), followed by centrifugation at 300×g for 5 min. The cells were washed three times with ice-cold blocking solution and filtered through a 50µm strainers. Subsequently, the cells were resuspended in an appropriate volume of blocking solution. To expedite FACS of endothelial cells, lineage depletion was performed using a lineage cell depletion kit (Miltenyi Biotec, 130-090-858), following the manufacturer's instructions. Lineage-depleted cells were resuspended in 0.5% BSA in PBS, and cell sorting was performed using a FACS Aria II cell sorter (BD Biosciences). Sorted GFP-tomato double-positive cells were collected in a 0.05% blocking buffer for scRNA-seq.
Instrument	FACS Aria II cell sorter (BD Bioscience)
Software	FACSDiva (8.0.2, BD Bioscience) and FlowJo (10.8.1) were used for sorting and analysis.
Cell population abundance	Total cell counts were measured with cell counter.
Gating strategy	Single viable cells were gated initally forward and side scatter.

☒ Tick this box to confirm that a figure exemplifying the gating strategy is provided in the Supplementary Information.
